# Rituximab resistant evans syndrome and autoimmunity in Schimke immuno-osseous dysplasia

**DOI:** 10.1186/1546-0096-9-27

**Published:** 2011-09-13

**Authors:** Jakub Zieg, Anna Krepelova, Alireza Baradaran-Heravi, Elena Levtchenko, Encarna Guillén-Navarro, Miroslava Balascakova, Martina Sukova, Tomas Seeman, Jiri Dusek, Nadezda Simankova, Tomas Rosik, Sylva Skalova, Jan Lebl, Cornelius F Boerkoel

**Affiliations:** 1 Department of Pediatrics, Second Faculty of Medicine, Charles University, University Hospital Motol, Prague, Czech Republic; 2 Department of Biology and Medical Genetics, Second Faculty of Medicine, Charles University, University Hospital Motol, Prague, Czech Republic; 3 Department of Medical Genetics, University of British Columbia, Vancouver, Canada; 4 Department of Pediatric Nephrology, University Hospitals Leuven, Leuven, Belgium; 5 Unidad de Genética Médica, Servicio de Pediatría, Hospital Universitario Virgen de La Arrixaca, Murcia, Spain; 6 Department of Pediatric Hematology and Oncology, Second Faculty of Medicine, Charles University, University Hospital Motol, Prague, Czech Republic; 7 Department of Pediatrics, Faculty of Medicine and University Hospital Hradec Králové, Charles University, Prague, Czech Republic

**Keywords:** Schimke immuno, osseous dysplasia, Unilateral renal agenesis, Nephrotic syndrome, Evans syndrome, Rituximab

## Abstract

Autoimmunity is often observed among individuals with primary immune deficiencies; however, the frequency and role of autoimmunity in Schimke immuno-osseous dysplasia (SIOD) has not been fully assessed. SIOD, which is caused by mutations of *SMARCAL1*, is a rare autosomal recessive disease with its prominent features being skeletal dysplasia, T cell deficiency, and renal failure. We present a child with severe SIOD who developed rituximab resistant Evans syndrome (ES). Consistent with observations in several other immunodeficiency disorders, a review of SIOD patients showed that approximately a fifth of SIOD patients have some features of autoimmune disease. To our best knowledge this case represents the first patient with SIOD and rituximab resistant ES and the first study of autoimmune disease in SIOD.

## Background

The immune system has evolved to clear pathogens efficiently and to tolerate self. The establishment and maintenance of self-tolerance is a requirement of adaptive immunity. To accomplish this, central tolerance removes self-reactive T cells during thymic development and peripheral tolerance represses self-reactive T cells that escape central tolerance checkpoints. Breakdown of either central or peripheral tolerance can lead to autoimmunity.

One such autoimmune disease is Evans syndrome (ES), which was first described in 1951 [1]. ES is defined by a combination (either simultaneously or sequentially) of autoimmune hemolytic anemia (AIHA) and idiopathic thrombocytopenic purpura (ITP) in the absence of an identifiable underlying pathology; ES can also include immune neutropenia [1]. Pediatric ES generally has a chronic course of frequent exacerbations and remissions and a mortality of 7-36%. Most patients respond to corticosteroids and/or intravenous immunoglobulins (IVIG), but relapse is frequent. Second-line therapies therefore include splenectomy or immunosuppressive drugs such as cyclosporine A (CsA), mycophenolate mophetil [1]. Recently, consistent with the hypothesis that ES arises from dysregulation of B cells, the monoclonal antibody against the B-cell antigen CD20, rituximab, has shown much promise for treatment of ES [1].

ES may be associated with other diseases or conditions such as systemic lupus erythematosus [2], lymphoproliferative disorders [3,4], or primary immunodeficiencies [5]. The primary immunodeficiencies, which are genetic disorders causing partial immune system dysfunction, are often characterised by aberrant inflammatory responses and autoimmunity [6]. Well-studied examples of this include autoimmune polyendocrinopathy candidiasis and ectodermal dystrophy (APECED), immunodysregulation, polyendocrinopathy, enteropathy, and X-linked inheritance (IPEX), autoimmune lymphoproliferative syndrome (ALPS), Wiskott-Aldrich syndrome (WAS), Omenn syndrome, C1q deficiency, interleukin-2 receptor alpha-chain deficiency and common variable immunodeficiency [7-11].

A less well-studied immunodeficiency is Schimke immunoosseous dysplasia (SIOD) [12]. SIOD, which was first described by Schimke *et al*. in 1971 [13], is a rare multisystem autosomal recessive disorder consisting of facial dysmorphism, spondyloepiphyseal dysplasia leading to dysproportionate growth failure, T-cell immunodeficiency and nephropathy characterised by steroid resistant nephrotic syndrome and frequently focal segmental glomerulosclerosis [14,15]. Additional features include ischemic cerebral attacks, migraine-like headaches, hematologic abnormalities of leucopenia, anemia and thrombocytopenia, enteropathy, hyperpigmented skin macules, unusual hair and microdontia [15-17]. The course of the disease varies from severe with intrauterine or early childhood onset and death in childhood [15,18,19] to milder disease with survival into adulthood [15,20]. For both severe and mild disease, the therapy is mainly symptomatic [15].

SIOD is caused by biallelic mutations in *SMARCAL1 *(SWI/SNF-related matrix-associated actin-dependent regulator of chromatin, subfamily-a-like-1), which encodes a DNA annealing helicase with homology to the SNF2 chromatin remodelling proteins [21]. The SMARCAL1 enzyme plays a role in the DNA stress response and regulates gene expression [22-27].

We report a child with severe SIOD and rituximab resistant Evans syndrome (ES) preceeding the bone marrow failure that can be associated with SIOD. Combined with prior reports of other autoimmune disorders among individuals with SIOD and upon review of our SIOD patient database, we conclude that the T cell immunodeficiency of SIOD compromises self-tolerance.

## Materials

### Patients

Patients referred to this study gave informed consent. The study was approved by the Institutional Review Boards of Baylor College of Medicine (Houston, TX USA), the Hospital for Sick Children (Toronto, ON Canada), and the University of British Columbia (Vancouver, BC Canada). Clinical data were obtained from questionnaires completed by the attending physician as well as medical summaries.

### Questionnaires

Physicians referring SIOD patients for molecular testing for SIOD complete a questionnaire on the patient being referred. Included in this questionnaire, is a question on whether the patient has an autoimmune disorder and if so to specify the problem, serology and therapy. In addition, there are specific questions asking if the patient had lymphocytopenia, neutropenia, anemia, or thrombocytopenia as well as the nature of the cytopenia and any effective therapies.

## Results

### Case

The proposita was a 4.5-year-old girl born to non-consanguineous parents by caesarean section at 36 weeks of gestation following a pregnancy complicated by intrauterine growth retardation. Her birth weight, length and head circumference were 1450 g (< 3^rd ^percentile), 39 cm (< 3^rd ^percentile) and 34 cm (25^th ^percentile), respectively. She did well in the newborn period with the exception of a self-limited thrombocytopenia. Her abdominal ultrasound detected left-sided unilateral renal agenesis (URA), but her voiding cystography was normal.

She was presented to our centre at the age of 3 years with nephrotic proteinuria of 1-2 g/m^2^/day without hematuria and a blood pressure of 90/65 mmHg (diastolic - 95^th ^percentile for height). On clinical examination, she had a high-pitched voice, low nasal bridge, short neck and trunk, disproportionate short stature, lumbar lordosis, protruding abdomen and numerous pigmented macules predominantly on her trunk. Her weight was 7.3 kg (< 3^rd ^percentile), and her length was 72 cm (< 3^rd ^percentile). She had normal neurologic development. Initial laboratory studies showed a normal blood count (WBC 5.2 cells per nl, HGB 13.8 g/dl, PLT 415 cells per nl), urea (3 mmol/l), creatinine (28 μmol/l), albumin (38.1 g/l), total protein (65.4 g/l), triglycerides (0.66 mmol/l), cholesterol (7 mmol/l), TSH (4.5 mIU/l), and free T4 (18.1 pmol/l). She also had normal growth hormone function tests. Immunological exam revealed deficiency of CD4^+ ^and CD8^+ ^cells and increased CD4^+^/CD8^+ ^ratio. Her laboratory results are summarised in tables 1 and 2.

**Table 1:** Evolution of laboratory values for the proposita

	Test results at the indicated ages					
**Age (years)**	**3^§^**	**3.5**	**4**	**4.5**	**5**	**5.5**
**Test** **(normal values)**						

WBC (cells per nl) (4-13)	6.5	6.1	4.1	2.8	1.2	1.1
HGB (g/dl) (11-15)	11.2	10.8	10.8	7	9.5	8.5
PLT (cells per nl) (140-440)	435	335	63	8	11	5
urea (mmol/l) (1.8-6.7)	3	5.2	7.1	10	6.6	10
creatinine (μmol/l)/GFR (ml/min/1.73 m^2^)	28/127	26/140	26/142	38/97	31/119	35/108
albumin (g/l) (35-53)	40.8	43.4	43.8	38	23.3	21.3
urine- protein/creatinine (mg/mmol) (< 20)	501	145	216	297	1321	1755

^§^While receiving prednisone (60 mg/m^2^/day)

**Table 2:** Evolution of the immune and autoimmune status of the proposita

	Test results at the indicated ages		
**Age (years)**	**3^§^**	**4**	**5^¶^**
**Test** **(normal values)**			

IgG (g/l) (5.53-10.20)	7.72	12.1	1.63
IgA (g/l) (0.33-0.91)	0.83	1.66	0.43
IgM (g/l) (0.47-1.67)	0.97	2.19	3.49
C3 (g/l) (0.83-2.25)	1.06	1.47	1.41
C4 (g/l) (0.14-0.35)	0.18	0.10	0.11
ANA	negative	negative	negative
ANCA	negative	negative	negative
ACLA (GPL/ml) (0.8-11)	7.0	6.3	4.3
ENA screening	negative	negative	NM
ATA IgA (U/ml) (0-10)	2.09	2.11	NM
CD4 absolute count x10^3^/ml (35-51%)	130 (12%)	200 (13%)	70 (50%)
CD8 absolute count x10^3^/ml (18-40%)	20 (1.8%)	170 (11%)	60 (44%)
CD4/CD8 (1-3%)	6.7%	1.2%	1.1%
CD19 absolute count x10^3^/ml (9-35%)	0.440 (45%)	530 (35%)	0 (0%)*

^§^While receiving prednisone (60 mg/m^2^/day)

^¶^Nephrotic syndrome worsened and proteinuria increased during the 5th year of life.

*After rituximab treatment.

Because of her immunodeficiency, she developed multiple infections. At the age of 3.5 years, she had *Mycoplasma pneumoniae *pneumonia with positive serology and required hospitalization for parenteral antibiotics. By the age of 4.5 years, she developed severe leucopenia and recurrent protracted infections including *Candida albicans *sepsis and Epstein Barr virus infection with fever and persistently high viral load associated with a decreased WBC.

At 4 years, she was hospitalized in status epilepticus. Cerebral magnetic resonance imaging showed multiple ischemic changes and narrowing of the middle cerebral artery bilaterally. The seizures responded to phenobarbital; however, she had additional cerebral infarcts and became triplegic with motoric aphasia.

To treat her multiple problems, we initated several therapies. For her nephrotic syndrome, we tried a 6 week course of prednisone (60 mg/m^2^/day); however, she did not respond and her subsequent renal biopsy showed focal segmental glomerulosclerosis. For treatment of her hypertension, we intially used the ACE inhibitor ramipril (2.5 mg daily) and subsequently the angiotensin receptor blocker losartan (12.5 mg daily); her arterial blood pressure declined to the normal range for her age and height. For treatment of her hypercholesterolemia, we introduced simvastatin therapy (5 mg daily), and her cholesterol level declined from 11 mmol/l back to 7.1 mmol/l. To control her recurrent infections, she was placed on prophylactic cotrimoxazole; however, the infections persisted and ultimately she died at 5.5 years from multiorgan failure secondary to *Enterobacter cloacae *sepsis.

Based on her renal failure, T-cell deficiency and dysmorphic features, we suspected the diagnosis of SIOD. Her skeletal radiographs also demonstrated the typical features including flattened capital femoral epiphyses, dysplastic acetabular fossae and marked thoracic kyphosis. To confirm the diagnosis molecularly, we sequenced PCR amplification products for the coding exons of *SMARCAL1*. This identified mutations c.2542G > T (p. Glu848X) in exon 17 and c.1439C > T (p.Pro480Leu) in exon 8; both of these mutations have been described previously in patients with SIOD [20,21]. Confirming compound heterozygosity, c.2542G > T was inherited from her mother and c.1439C > T was inherited from her father.

In addition to the above problems, the proposita developed ITP and anemia without splenomegaly in the latter half of her 5th year of life. The ITP was characterised by severe thrombocytopenia (8 cells per nl) and antiplatelet antibodies detectable by the immunobead assay. A year after her mycoplasma pneumonia, two months after onset of her ITP and before therapy with IVIG, she developed anemia (HGB 7 g/dl, reticulocytes 3.1%, LDH 7.61 μkat/l, bilirubin 11 μmol/l); this was accompanied by a direct antiglobulin test (DAT) that was positive for auto-antierythrocyte antibodies. She received one platelet transfusion and two red blood cell transfusions shortly after the diagnosis of her ITP and DAT-positive anemia, respectively.

Bone marrow aspirations performed at the onset of her ITP and again 3 months later revealed reduced platelet production despite normal numbers, size and morphology (nuclear separation) of megakaryocytes. Each also showed normal to hypercellular trilinear hematopoesis without dysplasia (myeloid precursors 47.2%, erythroid precursors 25%). Overall the bone marrow morphology was compatible with immune cytopenia and atypical for bone marrow failure. Based on this combination of findings and her clinical features, the proposita was diagnosed with ES.

Her ES responded poorly to standard therapies. It did not respond to corticosteroids or to IVIG alone. However, after a month of combination therapy with prednisone (2 mg/kg/day) and CyA (3-4 mg/kg/day) the platelet count and HGB level rose to the lower normal range. Unfortunately, the thrombocytopenia reoccured when the prednisone dose was weaned after 2 months; the relapse, which precipitated bleeding complications, was unresponsive to combined therapy with IVIG and high dose steroids as well as to rituximab (4 doses of 375 mg/m^2^). Throughout this time, the persistently DAT-positive anemia progressed such that she required 8 transfusions in the last months of her life.

Ten months after presenting with thrombocytopenia and eight months after the development of anemia, the proposita had the onset of progressive neutropenia. She did not have anti-neutrophil antibodies; therefore, we interpreted this as the onset of bone marrow failure. Concomitent with this she developed a severe coagulopathy and required four platelet transfusions.

### Autoimmune disease occurs commonly in SIOD patients

To ascertain whether the autoimmune disease observed in the proposita was unique to her or part of the clinical symptoms of SIOD, we sent questionnaires to 63 physicians of SIOD patients in whom we had identified *SMARCAL1 *mutations (Table 3), and 41 physicians reported checking for autoimmune disease in their patients. Among the 41 patients, 2 female and 6 male patients had one or more indications of autoimmune disease. These manifestations included thrombocytopenia, hemolytic anemia, enteropathy, and pericarditis with anti-cardiolipin antibodies. In one patient with thrombocytopenia, the autoimmune features resolved spontaneously, in another after bone marrow transplantation and in another after splenectomy. All other patients were successfully treated with immunosuppressive therapy: steroids, cyclophosphamide or IVIG.

**Table 3:** Autoimmune features identified in SIOD patients

Pedigree number	Gender	*SMARCAL1 *mutations	Autoimmune disease	Therapy	Reference
SD8	F	p.[L397RfsX39] +[*]	Autoimmune thrombocytopenia, autoimmune anemia	Thrombocytopenia resolved with steroids & IgG infusion.	[39]
SD23	M	p.[E848X] +[E848X]	Autoimmune bowel disease	Diarrhea and villous atrophy resolved with steroid treatment.	[16]
SD25	F	p.[R17X] +[Q34X]	Autoimmune thrombocytopenia	None; improved spontaneously.	
SD29	M	p.[R645PfsX16] +[D271_M288del]	Pericarditis, anti-cardiolipin antibodies	?	
SD49	M	p.[V641GfsX50] +[S774X]	Autoimmune bowel disease	Patient died before treatment.	
SD100	M	p.[E377Q]+[F279S]	Autoimmune thrombocytopenia	Thrombocytopenia resolved with splenectomy.	
SD102	M	p.[E848X]+[E848X]	Autoimmune bowel disease	?	
SD111	M	p.[E377Q]+[L531P]	Autoimmune thrombocytopenia and anemia	First episode improved spontaneously; at second episode patient died.	
SD140^¶^	F	p.[E848X]+[P480L]	Evans syndrome	Steroid, CSA and rituximab resistant; patient died.	

* No SMARCAL1 protein was detected in patient cells; therefore, the second allele is a null although a mutation was not detected by sequencing of the coding exons.

^¶^Proposita

## Discussion

As observed for SIOD [15,28,29], many other primary immune deficiencies are characterized by infections as well as a defect in self-tolerance. For example, APECED, ALPS and IPEX are defined by the occurrence of autoimmune diseases, whereas for other immune deficiencies the autoimmune manifestations are not as prominent [7-11]. Immunodeficiencies with less prominent autoimmune manifestations include common variable immunodeficiency [30,31], Good syndrome [32], hyper-IgM syndrome [33], WAS [34,35], and idiopathic CD4^+ ^lymphocytopenia [36]. About 22% of individuals with common variable immunodeficiency have autoimmune problems, and these include autoimmune cytopenias, pernicious anemia, thyroiditis, rheumatoid arthritis and vitiligo [30,31]. Approximately a quarter of individuals with hyper-IgM syndrome develop an autoimmune problem such as cytopenia, nephritis, enteropathy, hepatitis, arthritis, hypothyroidism or systemic lupus erythematosus (SLE) [33]. About half of individuals with WAS have autoimmune problems including neutropenia, arthritis, vasculitis, uveitis, enteropathy and nephritis [34,35]. Similarly, about 20-25% of those with idiopathic CD4^+ ^lymphocytopenia develop an autoimmune problem such as SLE, antiphospholipid syndrome, Grave's disease, colitis, thyroiditis and vitiligo [36]. Similar to these diseases, SIOD also has variable expression of autoimmune manifestations (Table 3).

The etiology of the autoimmune problems among SIOD patients remains undefined and no consistent inflammatory or serological markers have been reported [15]. As for the immunodeficiency, however, it likely arises from a dysfunction of SMARCAL1, the enzyme mutated in SIOD [21], within the lymphocytic lineages. Consistent with such a cell autonomous model, SMARCAL1 is highly expressed in the bone marrow lineages, and the limited data on one SIOD patient suggests that bone marrow transplantation can ameliorate the immunodeficiency [37,38].

## Conclusion

In conclusion, we report the first SIOD patient with autoimmune problems unresponsive to immunosuppression with steroids, CsA and rituximab. Additionally, we define the frequency and spectrum of autoimmune manifestions in SIOD.

## Consent

Written informed consent was obtained from the parents for publication of this case report and any accompanying images. A copy of the written consent is available for review by the Editor-in-Chief of this journal.

## Abbreviations

ACLA: anti-cardiolipin antibody; ACE: angiotensin-converting enzyme; ALPS: autoimmune lymphoproliferative syndrome; ANA: antinuclear antibody; ANCA: anti-neutrophil cytoplasmatic antibody; APECED: autoimmune polyendocrinopathy candidiasis and ectodermal dystrophy; ATA: anti-transglutaminase antibody; C1q: complement factor 1q; C3: complement factor 3; C4: complement factor 4; CD: cluster of differentiation protein; cm: centimeter; CsA: cyclosporine A; dl: deciliter; DNA: deoxyribonucleic acid; ENA: endonuclear antibody; ES: Evans syndrome; F: female; g: gram; HGB: hemoglobin; Ig: immunoglobulin; IPEX: immunodysregulation; polyendocrinopathy; enteropathy; and X-linked inheritance; IVIG: intravenous immunoglobulins; l: liter; LDH: lactate dehydrogenase; M: male; m^2^: square meter; mg: milligram; μmol: micro mole; mmol: millimole; nl: nanoliter; NM: not measured; p.: protein; PLT: platelet; SIOD: Schimke immuno-osseous dysplasia; SLE: systemic lupus erythematosus; SMARCAL1: SWI/SNF-related matrix associated actin-dependent regulator of chromatin; subfamily A-like protein 1; TSH: thyroid-stimulating hormone; URA: unilateral renal agenesis; WAS: Wiskott-Aldrich syndrome; WBC: white blood cell count.

## Competing interests

The authors declare that they have no competing interests.

## Authors' contributions

JZ wrote the initial manuscript draft. JD, TS and JL supervised the treatment of the patient and assisted in preparing the draft. MS, NS, SS and TR contributed in taking care of the patient and helped the drafting of the manuscript. AK, MB carried out molecular genetic testing and participated in the drafting of the manuscript. EL and EG-N contributed patient information not in the SIOD patient registry. ABH maintains the SIOD patient registry and complied the data in it for this report. CFB provided the SIOD patient registry and SIOD patient information. All authors critically reviewed and revised drafts. All authors read and approved the final manuscript.
